# (2,3,5,6-Tetra­fluoro­phenolato)[5,10,15,20-tetra­kis­(4-meth­oxy­phen­yl)porphyrinato]iron(III) cyclo­hexane monosolvate

**DOI:** 10.1107/S1600536813021880

**Published:** 2013-09-04

**Authors:** Nan Xu, Douglas R. Powell, George B. Richter-Addo

**Affiliations:** a Department of Chemistry and Biochemistry, University of Oklahoma, 101 Stephenson Pkwy, Norman, OK 73019, USA

## Abstract

The title compound, [Fe(C_6_HF_4_O)(C_48_H_36_N_4_O_4_)]·C_6_H_12_, represents a five-coordinate iron(III) porphyrin complex in a square-pyramidal geometry with a tetra­fluoro­phenolate anion as the axial ligand. The Fe^III^ atom is displaced by 0.364 (2) Å from the 24-atom mean plane of the porphyrinate ring towards the tetra­fluoro phenolate anion. The average Fe—N distance is 2.053 (2) Å and the Fe—O distance is 1.883 (2) Å. A porphyrin aryl H atom points in the general direction of the phenoxide ring. The mean plane separation between the 24-atom porphyrin planes of two adjacent porphyrin rings is ∼3.7 Å, and the lateral shift is ∼3.5 Å resu, ting in an Fe⋯Fe separation of 5.6167 (14) Å.

## Related literature

For the function and structure of catalase, see: Nicholls *et al.* (2001 ▶). For the structures of other related ferric pheno porphyrin derivatives, see: Chaudhary *et al.* (2010 ▶); Ueyama *et al.* (1998 ▶); Kanamori *et al.* (2005 ▶). For the typical geometry parameters for high-spin ferric porphyrin complexes, see: Scheidt & Reed (1981) ▶. For the synthesis of [(T(*p*-OMe)PP)Fe]_2_O, see: Helms *et al.* (1986 ▶).
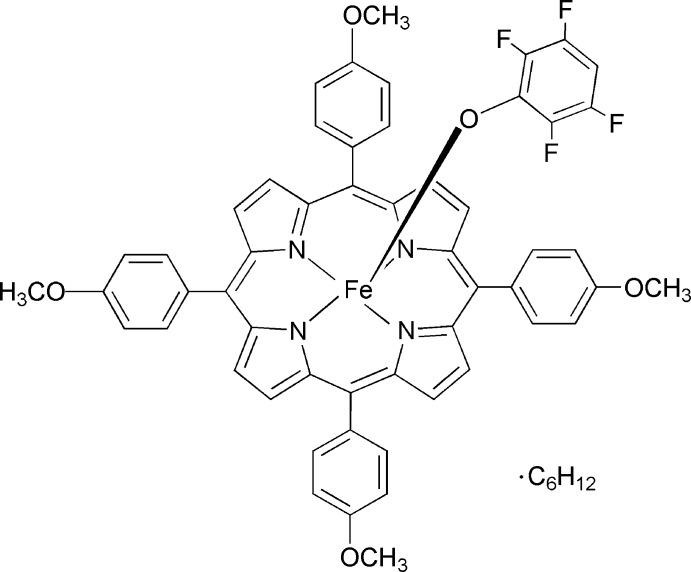


## Experimental

### 

#### Crystal data

[Fe(C_6_HF_4_O)(C_48_H_36_N_4_O_4_)]·C_6_H_12_
*M* *_r_* = 1037.88
Triclinic, 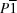

*a* = 10.294 (3) Å
*b* = 15.617 (5) Å
*c* = 16.082 (5) Å
α = 90.984 (7)°
β = 103.010 (8)°
γ = 103.869 (8)°
*V* = 2438.6 (13) Å^3^
*Z* = 2
Mo *K*α radiation
μ = 0.38 mm^−1^
*T* = 100 K
0.47 × 0.22 × 0.21 mm

#### Data collection

Bruker APEX diffractometer
Absorption correction: multi-scan (*SADABS*; Bruker, 2002 ▶) *T*_min_ = 0.841, *T*_max_ = 0.924
33926 measured reflections
12007 independent reflections
8303 reflections with *I* > 2σ(*I*)
*R*_int_ = 0.057
Standard reflections: ?

#### Refinement

*R*[*F* ^2^ > 2σ(*F* ^2^)] = 0.060
*wR*(*F* ^2^) = 0.159
*S* = 1.04
12007 reflections
671 parameters
69 restraints
H-atom parameters constrained
Δρ_max_ = 0.87 e Å^−3^
Δρ_min_ = −0.66 e Å^−3^

### 

Data collection: *SMART* (Bruker, 2007 ▶); cell refinement: *SAINT* (Bruker, 2007 ▶); data reduction: *SAINT*; program(s) used to solve structure: *SHELXL2013* (Sheldrick, 2008 ▶); program(s) used to refine structure: *SHELXL2013*; molecular graphics: *XP* in *SHELXTL* (Sheldrick, 2008 ▶); software used to prepare material for publication: *SHELXL2013*.

## Supplementary Material

Crystal structure: contains datablock(s) I, 10042m. DOI: 10.1107/S1600536813021880/ng5339sup1.cif

Structure factors: contains datablock(s) I. DOI: 10.1107/S1600536813021880/ng5339Isup2.hkl

Additional supplementary materials:  crystallographic information; 3D view; checkCIF report

## Figures and Tables

**Table 1:** Selected bond lengths (Å)

Fe1—N4	2.048 (2)
Fe1—N2	2.054 (2)
Fe1—N1	2.054 (2)
Fe1—N3	2.056 (2)

**Fig. 1.**
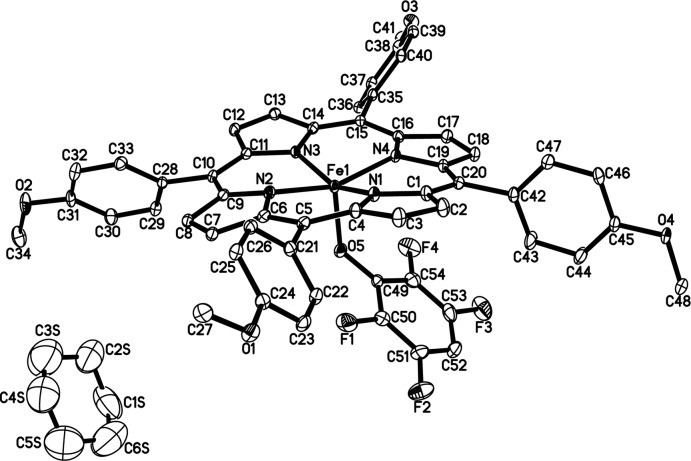
The molecular structure of (T(p-OMe)PP)Fe(OC6HF4).(C6H12) with displacement ellipsoids drawn at the 35% probability level. H atoms are omitted for clarity.

## supplementary crystallographic information

### 1. Comment

Synthetic metalloporphyrins have been studied intensively due to their potential to act as models for heme enzymes in biological systems. Catalase is a heme enzyme that catalyzes the decomposition of hydrogen peroxide to water and oxygen (Nicholls *et al.*, 2001). Heme catalase contains an active-site tyrosine, a phenolato type ligand, that binds to the heme iron center (Nicholls *et al.*, 2001). A number of iron phenolate porphyrin complexes has been prepared and structurally characterized (Chaudhary *et al.*, 2010, Ueyama *et al.*, 1998, Kanamori *et al.*, 2005). In this paper, we report the structure of (5,10,15,20-tetrakis(4-methoxyphenyl)porphyrinato)(tetrafluorophenolato)iron(III) with a cyclohexane monosolvate.

The molecular structure of (5,10,15,20-tetrakis(4-methoxyphenyl)porphyrinato)(2,3,5,6-tetrafluorophenolato)iron(III) is shown in Fig. 1. The porphyrin core of is saddle shaped. The iron atom is displaced by 0.364 (2) Å from the 24-atom mean porphyrin plane toward the tetrafluorophenolate anion. In addition, the Fe—N_p_ distances range from 2.048 (2) Å to 2.056 (2) Å, suggesting a high-spin ferric center (Scheidt & Reid, 1981). The Fe—O distance is 1.883 (2) Å which is typical of Fe—O bond distances in the iron phenolato porphyrin complexes reported previously. The Fe—O—C linkage shows a bent geometry with a bond angle of 130.46 (17)°.

### 2. Experimental

To a CH_2_Cl_2_ solution (20 ml) of [(T(*p*-OMe)PP)Fe]_2_O (Helms *et al.*, 1986) (0.030 g, 0.019 mmol) was added 2,3,5,6-tetrafluorophenol (0.051 g, 0.307 mmol) (purchased from Aldrich Chemical Company and used as received) under N_2_. After stirring for 1 h, the color of the solution changed from green to red. The solution was reduced to 2 ml under reduced pressure, and 10 ml hexane was added. The resulting black precipitation was collected by filtration and dried under vacuum. A suitable prism-shaped crystal was grown by slow evaporation of a CH_2_Cl_2_-cyclohexane (1:1) solution of the complex at room temperature under N_2_.

### 3. Refinement

The positions of H atoms bonded to carbons were initially determined by geometry and were refined using a riding model. H atoms bonded to N atoms and O atoms were located on a difference map, and their positions were refined independently. Non-hydrogen atoms were refined with anisotropic displacement parameters. Hydrogen atom displacement parameters were set to 1.2 (1.5 for methyl) times the isotropic equivalent displacement parameters of the bonded atoms. Carbon-bound H atoms were placed in calculated positions (aromatic 0.95, ethyl 0.99, and methyl 0.95 Angstroms) and were refined using a riding model. The C—C bond distances in the cyclohexane were restrained to 1.52 Angstroms and the 1–3 C···C distances were restrained to be equal. Rigid-bond restraints were also applied to the anisotropic displacement parameters of the solvent carbons.

### Crystal data

**Table:**

[Fe(C_6_HF_4_O)(C_48_H_36_N_4_O_4_)]·C_6_H_12_	*Z* = 2
*M**_r_* = 1037.88	*F*(000) = 1078
Triclinic, *P*1	*D*_x_ = 1.413 Mg m^−^^3^
*a* = 10.294 (3) Å	Mo *K*α radiation, λ = 0.71073 Å
*b* = 15.617 (5) Å	Cell parameters from 9754 reflections
*c* = 16.082 (5) Å	θ = 2.3–28.1°
α = 90.984 (7)°	µ = 0.38 mm^−^^1^
β = 103.010 (8)°	*T* = 100 K
γ = 103.869 (8)°	Prism, black
*V* = 2438.6 (13) Å^3^	0.47 × 0.22 × 0.21 mm

### Data collection

**Table:**

Bruker APEX diffractometer	8303 reflections with *I* > 2σ(*I*)
φ and ω scans	*R*_int_ = 0.057
Absorption correction: multi-scan (*SADABS*; Bruker, 2002)	θ_max_ = 28.3°, θ_min_ = 1.9°
*T*_min_ = 0.841, *T*_max_ = 0.924	*h* = −12→13
33926 measured reflections	*k* = −20→20
12007 independent reflections	*l* = −21→21

### Refinement

**Table:**

Refinement on *F*^2^	Primary atom site location: structure-invariant direct methods
Least-squares matrix: full	Secondary atom site location: difference Fourier map
*R*[*F*^2^ > 2σ(*F*^2^)] = 0.060	Hydrogen site location: inferred from neighbouring sites
*wR*(*F*^2^) = 0.159	H-atom parameters constrained
*S* = 1.04	*w* = 1/[σ^2^(*F*_o_^2^) + (0.070*P*)^2^ + 1.4*P*] where *P* = (*F*_o_^2^ + 2*F*_c_^2^)/3
12007 reflections	(Δ/σ)_max_ = 0.001
671 parameters	Δρ_max_ = 0.87 e Å^−^^3^
69 restraints	Δρ_min_ = −0.66 e Å^−^^3^

### Fractional atomic coordinates and isotropic or equivalent isotropic displacement parameters (Å^2^)

**Table:**

	*x*	*y*	*z*	*U*_iso_*/*U*_eq_	
Fe1	0.54850 (4)	0.40039 (2)	0.36835 (2)	0.01450 (11)	
F1	0.4734 (2)	0.37222 (14)	0.12137 (12)	0.0413 (5)	
F2	0.4990 (3)	0.25191 (16)	0.00941 (13)	0.0649 (7)	
F3	0.8499 (2)	0.18073 (15)	0.21702 (16)	0.0589 (7)	
F4	0.83646 (19)	0.30860 (14)	0.32666 (12)	0.0424 (5)	
O1	−0.2238 (2)	0.41310 (13)	−0.03430 (12)	0.0243 (5)	
O2	0.8053 (2)	1.00037 (13)	0.41908 (16)	0.0351 (6)	
O3	1.1901 (2)	0.33946 (13)	0.84395 (12)	0.0243 (5)	
O4	0.3294 (2)	−0.18471 (12)	0.21551 (12)	0.0255 (5)	
O5	0.6454 (2)	0.40630 (12)	0.28145 (12)	0.0255 (5)	
N1	0.3719 (2)	0.30765 (14)	0.30928 (14)	0.0173 (5)	
N2	0.4441 (2)	0.49621 (14)	0.33532 (13)	0.0156 (5)	
N3	0.6809 (2)	0.48904 (14)	0.46420 (13)	0.0144 (5)	
N4	0.6120 (2)	0.30102 (14)	0.43442 (14)	0.0160 (5)	
C1	0.3555 (3)	0.21711 (18)	0.30401 (17)	0.0187 (6)	
C2	0.2245 (3)	0.17497 (19)	0.24803 (18)	0.0242 (6)	
H2	0.1882	0.1132	0.2339	0.029*	
C3	0.1630 (3)	0.23938 (18)	0.21940 (18)	0.0237 (6)	
H3	0.0743	0.2312	0.1823	0.028*	
C4	0.2557 (3)	0.32252 (18)	0.25498 (17)	0.0181 (6)	
C5	0.2365 (3)	0.40538 (18)	0.23235 (16)	0.0175 (6)	
C6	0.3294 (3)	0.48624 (18)	0.26853 (16)	0.0171 (6)	
C7	0.3195 (3)	0.57118 (17)	0.23963 (17)	0.0180 (6)	
H7	0.2500	0.5825	0.1944	0.022*	
C8	0.4270 (3)	0.63249 (18)	0.28848 (16)	0.0170 (6)	
H8	0.4471	0.6946	0.2838	0.020*	
C9	0.5046 (3)	0.58593 (17)	0.34871 (16)	0.0157 (5)	
C10	0.6233 (3)	0.62673 (16)	0.41159 (16)	0.0155 (5)	
C11	0.6990 (3)	0.57991 (17)	0.46875 (16)	0.0154 (5)	
C12	0.8083 (3)	0.62018 (17)	0.54139 (16)	0.0176 (6)	
H12	0.8407	0.6816	0.5586	0.021*	
C13	0.8561 (3)	0.55420 (17)	0.58059 (16)	0.0178 (6)	
H13	0.9275	0.5607	0.6311	0.021*	
C14	0.7794 (3)	0.47207 (17)	0.53198 (16)	0.0157 (5)	
C15	0.8038 (3)	0.38896 (17)	0.54911 (16)	0.0145 (5)	
C16	0.7302 (3)	0.31050 (17)	0.49889 (16)	0.0161 (5)	
C17	0.7663 (3)	0.22693 (18)	0.50638 (17)	0.0196 (6)	
H17	0.8436	0.2157	0.5454	0.024*	
C18	0.6708 (3)	0.16726 (18)	0.44797 (17)	0.0191 (6)	
H18	0.6690	0.1068	0.4380	0.023*	
C19	0.5731 (3)	0.21268 (17)	0.40407 (17)	0.0174 (6)	
C20	0.4528 (3)	0.17299 (17)	0.34366 (16)	0.0174 (6)	
C21	0.1133 (3)	0.40780 (17)	0.16372 (17)	0.0181 (6)	
C22	0.0897 (3)	0.36430 (18)	0.08309 (17)	0.0205 (6)	
H22	0.1528	0.3324	0.0724	0.025*	
C23	−0.0231 (3)	0.36665 (18)	0.01868 (17)	0.0222 (6)	
H23	−0.0368	0.3367	−0.0357	0.027*	
C24	−0.1169 (3)	0.41300 (18)	0.03331 (17)	0.0193 (6)	
C25	−0.0956 (3)	0.45660 (18)	0.11327 (17)	0.0188 (6)	
H25	−0.1591	0.4881	0.1240	0.023*	
C26	0.0186 (3)	0.45390 (17)	0.17720 (17)	0.0186 (6)	
H26	0.0325	0.4842	0.2315	0.022*	
C27	−0.3165 (3)	0.4657 (2)	−0.02335 (19)	0.0256 (7)	
H27A	−0.3723	0.4381	0.0156	0.038*	
H27B	−0.3770	0.4696	−0.0789	0.038*	
H27C	−0.2635	0.5252	0.0008	0.038*	
C28	0.6737 (3)	0.72541 (17)	0.41580 (16)	0.0160 (6)	
C29	0.8035 (3)	0.76400 (18)	0.40272 (17)	0.0202 (6)	
H29	0.8613	0.7272	0.3941	0.024*	
C30	0.8507 (3)	0.85510 (19)	0.40196 (19)	0.0242 (6)	
H30	0.9387	0.8798	0.3914	0.029*	
C31	0.7689 (3)	0.90978 (18)	0.41667 (18)	0.0226 (6)	
C32	0.6398 (3)	0.87256 (19)	0.43091 (19)	0.0251 (7)	
H32	0.5833	0.9097	0.4409	0.030*	
C33	0.5928 (3)	0.78155 (18)	0.43067 (17)	0.0196 (6)	
H33	0.5044	0.7571	0.4408	0.024*	
C34	0.9249 (4)	1.0414 (2)	0.3893 (2)	0.0401 (9)	
H34A	1.0068	1.0298	0.4272	0.060*	
H34B	0.9344	1.1054	0.3894	0.060*	
H34C	0.9154	1.0171	0.3310	0.060*	
C35	0.9110 (3)	0.38021 (17)	0.62544 (16)	0.0159 (5)	
C36	1.0483 (3)	0.42617 (17)	0.64082 (16)	0.0172 (6)	
H36	1.0771	0.4669	0.6014	0.021*	
C37	1.1450 (3)	0.41403 (18)	0.71251 (17)	0.0181 (6)	
H37	1.2385	0.4462	0.7217	0.022*	
C38	1.1040 (3)	0.35481 (18)	0.77047 (17)	0.0183 (6)	
C39	0.9663 (3)	0.30805 (17)	0.75673 (17)	0.0175 (6)	
H39	0.9376	0.2679	0.7966	0.021*	
C40	0.8725 (3)	0.32036 (17)	0.68520 (16)	0.0161 (5)	
H40	0.7793	0.2876	0.6759	0.019*	
C41	1.3346 (3)	0.3732 (2)	0.8523 (2)	0.0316 (7)	
H41A	1.3606	0.3515	0.8025	0.047*	
H41B	1.3852	0.3532	0.9044	0.047*	
H41C	1.3572	0.4379	0.8559	0.047*	
C42	0.4271 (3)	0.07705 (17)	0.31487 (17)	0.0180 (6)	
C43	0.4813 (4)	0.0550 (2)	0.2500 (2)	0.0452 (11)	
H43	0.5405	0.1003	0.2277	0.054*	
C44	0.4528 (4)	−0.0317 (2)	0.2153 (2)	0.0432 (10)	
H44	0.4926	−0.0450	0.1703	0.052*	
C45	0.3675 (3)	−0.09726 (17)	0.24639 (17)	0.0187 (6)	
C46	0.3129 (4)	−0.07695 (19)	0.3128 (2)	0.0338 (8)	
H46	0.2541	−0.1224	0.3353	0.041*	
C47	0.3439 (4)	0.00976 (19)	0.3468 (2)	0.0322 (8)	
H47	0.3068	0.0229	0.3931	0.039*	
C48	0.3723 (4)	−0.2052 (2)	0.1411 (2)	0.0355 (8)	
H48A	0.3400	−0.1694	0.0952	0.053*	
H48B	0.3334	−0.2681	0.1226	0.053*	
H48C	0.4730	−0.1922	0.1541	0.053*	
C49	0.6532 (3)	0.34337 (18)	0.22824 (17)	0.0195 (6)	
C50	0.5682 (3)	0.3249 (2)	0.14612 (19)	0.0265 (7)	
C51	0.5817 (4)	0.2627 (2)	0.0895 (2)	0.0373 (8)	
C52	0.6752 (4)	0.2133 (2)	0.1113 (2)	0.0397 (9)	
H52	0.6826	0.1696	0.0719	0.048*	
C53	0.7571 (4)	0.2293 (2)	0.1916 (2)	0.0350 (8)	
C54	0.7490 (3)	0.2932 (2)	0.24924 (19)	0.0268 (7)	
C1S	0.9771 (6)	0.8891 (5)	0.0965 (4)	0.127 (2)	
H1S1	1.0226	0.8403	0.1106	0.152*	
H1S2	1.0481	0.9416	0.0888	0.152*	
C2S	0.9200 (8)	0.9095 (5)	0.1691 (4)	0.145 (3)	
H2S1	0.9972	0.9401	0.2168	0.174*	
H2S2	0.8739	0.8532	0.1890	0.174*	
C3S	0.8180 (9)	0.9665 (6)	0.1485 (4)	0.173 (3)	
H3S1	0.7669	0.9622	0.1941	0.208*	
H3S2	0.8708	1.0288	0.1507	0.208*	
C4S	0.7174 (6)	0.9457 (5)	0.0658 (4)	0.132 (2)	
H4S1	0.6791	0.9975	0.0519	0.159*	
H4S2	0.6406	0.8960	0.0718	0.159*	
C5S	0.7717 (8)	0.9213 (6)	−0.0085 (4)	0.150 (3)	
H5S1	0.6930	0.8907	−0.0553	0.180*	
H5S2	0.8196	0.9762	−0.0299	0.180*	
C6S	0.8694 (9)	0.8628 (6)	0.0135 (4)	0.186 (4)	
H6S1	0.9169	0.8618	−0.0335	0.223*	
H6S2	0.8149	0.8018	0.0160	0.223*	

### Atomic displacement parameters (Å^2^)

**Table:**

	*U*^11^	*U*^22^	*U*^33^	*U*^12^	*U*^13^	*U*^23^
Fe1	0.0163 (2)	0.01043 (19)	0.01492 (19)	0.00252 (15)	0.00085 (15)	−0.00100 (14)
F1	0.0424 (12)	0.0535 (13)	0.0327 (10)	0.0241 (10)	0.0049 (9)	0.0109 (9)
F2	0.0936 (19)	0.0673 (16)	0.0233 (10)	0.0151 (14)	−0.0010 (11)	−0.0129 (10)
F3	0.0588 (15)	0.0543 (15)	0.0928 (18)	0.0405 (12)	0.0473 (14)	0.0322 (13)
F4	0.0237 (10)	0.0589 (14)	0.0405 (11)	0.0075 (10)	0.0015 (9)	0.0105 (10)
O1	0.0211 (11)	0.0280 (11)	0.0200 (10)	0.0091 (9)	−0.0056 (8)	−0.0050 (8)
O2	0.0315 (13)	0.0111 (10)	0.0604 (15)	0.0033 (9)	0.0087 (11)	0.0018 (10)
O3	0.0172 (10)	0.0304 (12)	0.0213 (10)	0.0053 (9)	−0.0031 (8)	0.0024 (8)
O4	0.0387 (13)	0.0097 (9)	0.0264 (11)	0.0017 (9)	0.0094 (9)	−0.0043 (8)
O5	0.0352 (12)	0.0143 (10)	0.0266 (11)	−0.0006 (9)	0.0142 (9)	−0.0026 (8)
N1	0.0175 (12)	0.0130 (11)	0.0200 (11)	0.0039 (9)	0.0014 (9)	0.0000 (9)
N2	0.0151 (12)	0.0142 (11)	0.0160 (10)	0.0039 (9)	0.0002 (9)	−0.0014 (8)
N3	0.0169 (12)	0.0098 (11)	0.0152 (10)	0.0030 (9)	0.0019 (9)	0.0005 (8)
N4	0.0185 (12)	0.0107 (11)	0.0177 (11)	0.0038 (9)	0.0021 (9)	−0.0009 (8)
C1	0.0188 (15)	0.0134 (13)	0.0217 (13)	0.0027 (11)	0.0017 (11)	0.0004 (10)
C2	0.0228 (16)	0.0148 (14)	0.0274 (15)	−0.0002 (12)	−0.0044 (12)	−0.0013 (11)
C3	0.0191 (15)	0.0176 (15)	0.0280 (15)	0.0023 (12)	−0.0046 (12)	−0.0019 (12)
C4	0.0159 (14)	0.0150 (13)	0.0216 (13)	0.0032 (11)	0.0016 (11)	0.0000 (10)
C5	0.0175 (14)	0.0174 (14)	0.0170 (13)	0.0046 (11)	0.0025 (11)	−0.0019 (10)
C6	0.0180 (14)	0.0172 (14)	0.0159 (12)	0.0050 (11)	0.0033 (11)	−0.0018 (10)
C7	0.0218 (15)	0.0164 (14)	0.0164 (12)	0.0081 (12)	0.0021 (11)	−0.0005 (10)
C8	0.0203 (15)	0.0138 (13)	0.0174 (13)	0.0057 (11)	0.0037 (11)	−0.0002 (10)
C9	0.0178 (14)	0.0148 (13)	0.0151 (12)	0.0056 (11)	0.0039 (11)	−0.0009 (10)
C10	0.0199 (14)	0.0100 (12)	0.0169 (12)	0.0040 (11)	0.0048 (11)	−0.0018 (10)
C11	0.0176 (14)	0.0129 (13)	0.0157 (12)	0.0032 (11)	0.0044 (11)	−0.0010 (10)
C12	0.0205 (15)	0.0122 (13)	0.0173 (13)	0.0016 (11)	0.0018 (11)	−0.0028 (10)
C13	0.0201 (15)	0.0164 (14)	0.0149 (12)	0.0040 (11)	0.0010 (11)	−0.0003 (10)
C14	0.0151 (13)	0.0136 (13)	0.0175 (12)	0.0023 (11)	0.0037 (11)	−0.0025 (10)
C15	0.0161 (13)	0.0128 (13)	0.0148 (12)	0.0038 (11)	0.0039 (10)	0.0011 (10)
C16	0.0172 (14)	0.0147 (13)	0.0153 (12)	0.0043 (11)	0.0015 (11)	0.0013 (10)
C17	0.0217 (15)	0.0166 (14)	0.0199 (13)	0.0066 (12)	0.0018 (12)	−0.0003 (11)
C18	0.0216 (15)	0.0127 (13)	0.0218 (13)	0.0052 (11)	0.0015 (12)	−0.0011 (10)
C19	0.0220 (15)	0.0111 (13)	0.0188 (13)	0.0041 (11)	0.0045 (11)	0.0006 (10)
C20	0.0224 (15)	0.0120 (13)	0.0166 (12)	0.0023 (11)	0.0042 (11)	−0.0007 (10)
C21	0.0187 (14)	0.0144 (13)	0.0177 (13)	0.0020 (11)	−0.0004 (11)	0.0001 (10)
C22	0.0215 (15)	0.0165 (14)	0.0229 (14)	0.0066 (12)	0.0024 (12)	−0.0024 (11)
C23	0.0264 (16)	0.0183 (14)	0.0183 (13)	0.0045 (12)	−0.0004 (12)	−0.0069 (11)
C24	0.0183 (15)	0.0174 (14)	0.0182 (13)	0.0022 (11)	−0.0011 (11)	−0.0010 (10)
C25	0.0180 (14)	0.0167 (14)	0.0217 (13)	0.0063 (11)	0.0029 (11)	−0.0012 (11)
C26	0.0220 (15)	0.0160 (14)	0.0163 (13)	0.0048 (11)	0.0018 (11)	−0.0010 (10)
C27	0.0198 (16)	0.0319 (17)	0.0238 (15)	0.0092 (13)	−0.0004 (12)	0.0018 (12)
C28	0.0220 (15)	0.0121 (13)	0.0137 (12)	0.0057 (11)	0.0023 (11)	0.0004 (10)
C29	0.0208 (15)	0.0149 (14)	0.0241 (14)	0.0056 (11)	0.0029 (12)	−0.0015 (11)
C30	0.0205 (15)	0.0175 (15)	0.0326 (16)	0.0014 (12)	0.0055 (13)	0.0013 (12)
C31	0.0267 (16)	0.0093 (13)	0.0287 (15)	0.0041 (12)	0.0008 (13)	0.0002 (11)
C32	0.0243 (16)	0.0166 (14)	0.0345 (16)	0.0089 (12)	0.0036 (13)	−0.0038 (12)
C33	0.0191 (15)	0.0173 (14)	0.0218 (13)	0.0027 (12)	0.0057 (11)	−0.0010 (11)
C34	0.043 (2)	0.0154 (16)	0.055 (2)	−0.0035 (15)	0.0107 (18)	0.0054 (15)
C35	0.0180 (14)	0.0115 (13)	0.0166 (12)	0.0034 (11)	0.0016 (11)	−0.0019 (10)
C36	0.0211 (15)	0.0130 (13)	0.0175 (13)	0.0036 (11)	0.0053 (11)	−0.0003 (10)
C37	0.0143 (14)	0.0150 (13)	0.0240 (14)	0.0032 (11)	0.0033 (11)	−0.0030 (11)
C38	0.0179 (14)	0.0183 (14)	0.0173 (13)	0.0076 (11)	−0.0014 (11)	−0.0024 (10)
C39	0.0190 (14)	0.0133 (13)	0.0200 (13)	0.0034 (11)	0.0048 (11)	0.0018 (10)
C40	0.0143 (13)	0.0127 (13)	0.0193 (13)	0.0016 (11)	0.0018 (11)	−0.0010 (10)
C41	0.0176 (16)	0.043 (2)	0.0298 (16)	0.0080 (14)	−0.0043 (13)	0.0031 (14)
C42	0.0191 (14)	0.0117 (13)	0.0196 (13)	0.0025 (11)	−0.0014 (11)	−0.0015 (10)
C43	0.068 (3)	0.0129 (15)	0.061 (2)	−0.0072 (16)	0.047 (2)	−0.0058 (15)
C44	0.069 (3)	0.0175 (16)	0.051 (2)	−0.0002 (17)	0.044 (2)	−0.0074 (15)
C45	0.0209 (15)	0.0115 (13)	0.0206 (13)	0.0039 (11)	−0.0010 (11)	−0.0021 (10)
C46	0.052 (2)	0.0112 (14)	0.0396 (18)	−0.0016 (14)	0.0232 (17)	0.0018 (13)
C47	0.051 (2)	0.0161 (15)	0.0321 (17)	0.0023 (15)	0.0231 (16)	−0.0020 (12)
C48	0.064 (2)	0.0159 (15)	0.0278 (16)	0.0072 (16)	0.0163 (16)	−0.0042 (12)
C49	0.0224 (15)	0.0132 (13)	0.0236 (14)	0.0003 (11)	0.0114 (12)	0.0009 (11)
C50	0.0304 (17)	0.0265 (16)	0.0240 (15)	0.0086 (14)	0.0077 (13)	0.0042 (12)
C51	0.053 (2)	0.0341 (19)	0.0209 (15)	0.0047 (17)	0.0081 (15)	−0.0039 (13)
C52	0.063 (3)	0.0246 (17)	0.042 (2)	0.0129 (17)	0.0312 (19)	−0.0009 (15)
C53	0.039 (2)	0.0263 (18)	0.054 (2)	0.0192 (15)	0.0277 (18)	0.0165 (15)
C54	0.0227 (16)	0.0279 (17)	0.0304 (16)	0.0032 (13)	0.0104 (13)	0.0052 (13)
C1S	0.130 (5)	0.112 (5)	0.166 (5)	0.043 (4)	0.069 (4)	0.076 (4)
C2S	0.204 (6)	0.117 (5)	0.126 (5)	0.071 (5)	0.032 (4)	−0.010 (4)
C3S	0.199 (7)	0.182 (7)	0.157 (5)	0.093 (5)	0.031 (5)	−0.007 (5)
C4S	0.118 (5)	0.122 (5)	0.165 (5)	0.028 (4)	0.052 (4)	0.010 (4)
C5S	0.134 (5)	0.176 (6)	0.132 (5)	0.029 (5)	0.026 (4)	0.011 (5)
C6S	0.249 (7)	0.186 (7)	0.151 (5)	0.095 (5)	0.063 (5)	−0.027 (5)

### Geometric parameters (Å, º)

**Table:**

Fe1—O5	1.883 (2)	C27—H27A	0.9800
Fe1—N4	2.048 (2)	C27—H27B	0.9800
Fe1—N2	2.054 (2)	C27—H27C	0.9800
Fe1—N1	2.054 (2)	C28—C29	1.392 (4)
Fe1—N3	2.056 (2)	C28—C33	1.397 (4)
F1—C50	1.356 (4)	C29—C30	1.390 (4)
F2—C51	1.358 (4)	C29—H29	0.9500
F3—C53	1.355 (4)	C30—C31	1.386 (4)
F4—C54	1.342 (4)	C30—H30	0.9500
O1—C24	1.362 (3)	C31—C32	1.388 (4)
O1—C27	1.436 (3)	C32—C33	1.387 (4)
O2—C31	1.372 (3)	C32—H32	0.9500
O2—C34	1.433 (4)	C33—H33	0.9500
O3—C38	1.371 (3)	C34—H34A	0.9800
O3—C41	1.428 (4)	C34—H34B	0.9800
O4—C45	1.378 (3)	C34—H34C	0.9800
O4—C48	1.422 (3)	C35—C36	1.387 (4)
O5—C49	1.321 (3)	C35—C40	1.407 (4)
N1—C1	1.382 (3)	C36—C37	1.392 (4)
N1—C4	1.384 (4)	C36—H36	0.9500
N2—C6	1.381 (3)	C37—C38	1.386 (4)
N2—C9	1.382 (3)	C37—H37	0.9500
N3—C11	1.385 (3)	C38—C39	1.396 (4)
N3—C14	1.388 (3)	C39—C40	1.375 (4)
N4—C16	1.384 (3)	C39—H39	0.9500
N4—C19	1.388 (3)	C40—H40	0.9500
C1—C20	1.394 (4)	C41—H41A	0.9800
C1—C2	1.438 (4)	C41—H41B	0.9800
C2—C3	1.347 (4)	C41—H41C	0.9800
C2—H2	0.9500	C42—C43	1.364 (4)
C3—C4	1.435 (4)	C42—C47	1.374 (4)
C3—H3	0.9500	C43—C44	1.393 (4)
C4—C5	1.397 (4)	C43—H43	0.9500
C5—C6	1.412 (4)	C44—C45	1.361 (4)
C5—C21	1.490 (4)	C44—H44	0.9500
C6—C7	1.432 (4)	C45—C46	1.379 (4)
C7—C8	1.354 (4)	C46—C47	1.387 (4)
C7—H7	0.9500	C46—H46	0.9500
C8—C9	1.435 (4)	C47—H47	0.9500
C8—H8	0.9500	C48—H48A	0.9800
C9—C10	1.404 (4)	C48—H48B	0.9800
C10—C11	1.400 (4)	C48—H48C	0.9800
C10—C28	1.499 (4)	C49—C54	1.389 (4)
C11—C12	1.439 (4)	C49—C50	1.392 (4)
C12—C13	1.352 (4)	C50—C51	1.371 (4)
C12—H12	0.9500	C51—C52	1.364 (5)
C13—C14	1.443 (3)	C52—C53	1.356 (5)
C13—H13	0.9500	C52—H52	0.9500
C14—C15	1.400 (4)	C53—C54	1.380 (4)
C15—C16	1.405 (3)	C1S—C2S	1.481 (5)
C15—C35	1.487 (4)	C1S—C6S	1.509 (5)
C16—C17	1.440 (4)	C1S—H1S1	0.9900
C17—C18	1.354 (4)	C1S—H1S2	0.9900
C17—H17	0.9500	C2S—C3S	1.519 (5)
C18—C19	1.433 (4)	C2S—H2S1	0.9900
C18—H18	0.9500	C2S—H2S2	0.9900
C19—C20	1.389 (4)	C3S—C4S	1.467 (5)
C20—C42	1.503 (4)	C3S—H3S1	0.9900
C21—C26	1.394 (4)	C3S—H3S2	0.9900
C21—C22	1.397 (4)	C4S—C5S	1.507 (5)
C22—C23	1.379 (4)	C4S—H4S1	0.9900
C22—H22	0.9500	C4S—H4S2	0.9900
C23—C24	1.394 (4)	C5S—C6S	1.504 (5)
C23—H23	0.9500	C5S—H5S1	0.9900
C24—C25	1.392 (4)	C5S—H5S2	0.9900
C25—C26	1.386 (4)	C6S—H6S1	0.9900
C25—H25	0.9500	C6S—H6S2	0.9900
C26—H26	0.9500		
			
O5—Fe1—N4	100.32 (9)	C29—C30—H30	120.1
O5—Fe1—N2	100.55 (9)	O2—C31—C30	125.0 (3)
N4—Fe1—N2	159.11 (9)	O2—C31—C32	115.6 (3)
O5—Fe1—N1	99.31 (9)	C30—C31—C32	119.4 (3)
N4—Fe1—N1	87.60 (9)	C33—C32—C31	120.5 (3)
N2—Fe1—N1	88.17 (9)	C33—C32—H32	119.7
O5—Fe1—N3	103.67 (9)	C31—C32—H32	119.7
N4—Fe1—N3	88.18 (9)	C32—C33—C28	120.9 (3)
N2—Fe1—N3	87.76 (9)	C32—C33—H33	119.6
N1—Fe1—N3	157.02 (9)	C28—C33—H33	119.6
C24—O1—C27	117.5 (2)	O2—C34—H34A	109.5
C31—O2—C34	117.8 (2)	O2—C34—H34B	109.5
C38—O3—C41	117.1 (2)	H34A—C34—H34B	109.5
C45—O4—C48	116.8 (2)	O2—C34—H34C	109.5
C49—O5—Fe1	130.46 (17)	H34A—C34—H34C	109.5
C1—N1—C4	106.0 (2)	H34B—C34—H34C	109.5
C1—N1—Fe1	125.89 (19)	C36—C35—C40	117.5 (2)
C4—N1—Fe1	127.28 (18)	C36—C35—C15	124.1 (2)
C6—N2—C9	106.2 (2)	C40—C35—C15	118.5 (2)
C6—N2—Fe1	124.70 (17)	C35—C36—C37	121.6 (3)
C9—N2—Fe1	123.93 (18)	C35—C36—H36	119.2
C11—N3—C14	106.0 (2)	C37—C36—H36	119.2
C11—N3—Fe1	126.15 (17)	C38—C37—C36	119.6 (3)
C14—N3—Fe1	127.56 (17)	C38—C37—H37	120.2
C16—N4—C19	106.0 (2)	C36—C37—H37	120.2
C16—N4—Fe1	126.28 (17)	O3—C38—C37	124.3 (3)
C19—N4—Fe1	124.43 (18)	O3—C38—C39	115.8 (2)
N1—C1—C20	125.6 (2)	C37—C38—C39	119.9 (3)
N1—C1—C2	109.6 (2)	C40—C39—C38	119.7 (3)
C20—C1—C2	124.7 (2)	C40—C39—H39	120.2
C3—C2—C1	107.2 (2)	C38—C39—H39	120.2
C3—C2—H2	126.4	C39—C40—C35	121.7 (3)
C1—C2—H2	126.4	C39—C40—H40	119.2
C2—C3—C4	107.7 (3)	C35—C40—H40	119.2
C2—C3—H3	126.2	O3—C41—H41A	109.5
C4—C3—H3	126.2	O3—C41—H41B	109.5
N1—C4—C5	125.4 (2)	H41A—C41—H41B	109.5
N1—C4—C3	109.4 (2)	O3—C41—H41C	109.5
C5—C4—C3	124.9 (3)	H41A—C41—H41C	109.5
C4—C5—C6	123.7 (3)	H41B—C41—H41C	109.5
C4—C5—C21	117.7 (2)	C43—C42—C47	117.4 (3)
C6—C5—C21	118.5 (2)	C43—C42—C20	119.1 (3)
N2—C6—C5	126.1 (3)	C47—C42—C20	123.4 (2)
N2—C6—C7	109.5 (2)	C42—C43—C44	122.3 (3)
C5—C6—C7	124.4 (3)	C42—C43—H43	118.8
C8—C7—C6	107.6 (2)	C44—C43—H43	118.8
C8—C7—H7	126.2	C45—C44—C43	119.5 (3)
C6—C7—H7	126.2	C45—C44—H44	120.3
C7—C8—C9	107.1 (2)	C43—C44—H44	120.3
C7—C8—H8	126.5	C44—C45—O4	124.4 (3)
C9—C8—H8	126.5	C44—C45—C46	119.4 (3)
N2—C9—C10	126.0 (2)	O4—C45—C46	116.2 (2)
N2—C9—C8	109.7 (2)	C45—C46—C47	120.1 (3)
C10—C9—C8	124.4 (2)	C45—C46—H46	120.0
C11—C10—C9	123.4 (2)	C47—C46—H46	120.0
C11—C10—C28	118.5 (2)	C42—C47—C46	121.3 (3)
C9—C10—C28	118.1 (2)	C42—C47—H47	119.4
N3—C11—C10	125.7 (2)	C46—C47—H47	119.4
N3—C11—C12	110.0 (2)	O4—C48—H48A	109.5
C10—C11—C12	124.4 (2)	O4—C48—H48B	109.5
C13—C12—C11	107.1 (2)	H48A—C48—H48B	109.5
C13—C12—H12	126.5	O4—C48—H48C	109.5
C11—C12—H12	126.5	H48A—C48—H48C	109.5
C12—C13—C14	107.8 (2)	H48B—C48—H48C	109.5
C12—C13—H13	126.1	O5—C49—C54	122.5 (3)
C14—C13—H13	126.1	O5—C49—C50	121.9 (3)
N3—C14—C15	125.6 (2)	C54—C49—C50	115.6 (3)
N3—C14—C13	109.2 (2)	F1—C50—C51	119.8 (3)
C15—C14—C13	125.2 (2)	F1—C50—C49	118.5 (3)
C14—C15—C16	123.9 (2)	C51—C50—C49	121.7 (3)
C14—C15—C35	119.8 (2)	F2—C51—C52	120.1 (3)
C16—C15—C35	116.3 (2)	F2—C51—C50	117.8 (3)
N4—C16—C15	125.7 (2)	C52—C51—C50	122.1 (3)
N4—C16—C17	109.2 (2)	C53—C52—C51	116.9 (3)
C15—C16—C17	125.1 (2)	C53—C52—H52	121.5
C18—C17—C16	107.9 (3)	C51—C52—H52	121.5
C18—C17—H17	126.1	F3—C53—C52	119.3 (3)
C16—C17—H17	126.1	F3—C53—C54	118.3 (3)
C17—C18—C19	106.9 (2)	C52—C53—C54	122.4 (3)
C17—C18—H18	126.5	F4—C54—C53	119.8 (3)
C19—C18—H18	126.5	F4—C54—C49	119.0 (3)
N4—C19—C20	125.0 (3)	C53—C54—C49	121.3 (3)
N4—C19—C18	110.0 (2)	C2S—C1S—C6S	113.2 (5)
C20—C19—C18	124.9 (2)	C2S—C1S—H1S1	108.9
C19—C20—C1	124.5 (2)	C6S—C1S—H1S1	108.9
C19—C20—C42	118.8 (2)	C2S—C1S—H1S2	108.9
C1—C20—C42	116.7 (2)	C6S—C1S—H1S2	108.9
C26—C21—C22	117.7 (2)	H1S1—C1S—H1S2	107.8
C26—C21—C5	121.5 (2)	C1S—C2S—C3S	114.6 (4)
C22—C21—C5	120.8 (2)	C1S—C2S—H2S1	108.6
C23—C22—C21	121.5 (3)	C3S—C2S—H2S1	108.6
C23—C22—H22	119.3	C1S—C2S—H2S2	108.6
C21—C22—H22	119.3	C3S—C2S—H2S2	108.6
C22—C23—C24	120.1 (2)	H2S1—C2S—H2S2	107.6
C22—C23—H23	120.0	C4S—C3S—C2S	116.8 (5)
C24—C23—H23	120.0	C4S—C3S—H3S1	108.1
O1—C24—C25	124.5 (3)	C2S—C3S—H3S1	108.1
O1—C24—C23	116.1 (2)	C4S—C3S—H3S2	108.1
C25—C24—C23	119.4 (3)	C2S—C3S—H3S2	108.1
C26—C25—C24	119.8 (3)	H3S1—C3S—H3S2	107.3
C26—C25—H25	120.1	C3S—C4S—C5S	115.6 (4)
C24—C25—H25	120.1	C3S—C4S—H4S1	108.4
C25—C26—C21	121.5 (2)	C5S—C4S—H4S1	108.4
C25—C26—H26	119.2	C3S—C4S—H4S2	108.4
C21—C26—H26	119.2	C5S—C4S—H4S2	108.4
O1—C27—H27A	109.5	H4S1—C4S—H4S2	107.4
O1—C27—H27B	109.5	C6S—C5S—C4S	113.7 (5)
H27A—C27—H27B	109.5	C6S—C5S—H5S1	108.8
O1—C27—H27C	109.5	C4S—C5S—H5S1	108.8
H27A—C27—H27C	109.5	C6S—C5S—H5S2	108.8
H27B—C27—H27C	109.5	C4S—C5S—H5S2	108.8
C29—C28—C33	117.8 (2)	H5S1—C5S—H5S2	107.7
C29—C28—C10	120.2 (2)	C5S—C6S—C1S	115.7 (5)
C33—C28—C10	122.1 (2)	C5S—C6S—H6S1	108.4
C30—C29—C28	121.7 (3)	C1S—C6S—H6S1	108.4
C30—C29—H29	119.2	C5S—C6S—H6S2	108.4
C28—C29—H29	119.2	C1S—C6S—H6S2	108.4
C31—C30—C29	119.8 (3)	H6S1—C6S—H6S2	107.4
C31—C30—H30	120.1		
			
N4—Fe1—O5—C49	51.2 (3)	C4—C5—C21—C22	−55.8 (4)
N2—Fe1—O5—C49	−127.9 (3)	C6—C5—C21—C22	121.9 (3)
N1—Fe1—O5—C49	−38.0 (3)	C26—C21—C22—C23	0.1 (4)
N3—Fe1—O5—C49	141.8 (3)	C5—C21—C22—C23	−179.4 (3)
C4—N1—C1—C20	175.2 (3)	C21—C22—C23—C24	−0.2 (4)
Fe1—N1—C1—C20	5.3 (4)	C27—O1—C24—C25	3.4 (4)
C4—N1—C1—C2	−2.2 (3)	C27—O1—C24—C23	−175.4 (3)
Fe1—N1—C1—C2	−172.12 (18)	C22—C23—C24—O1	178.9 (3)
N1—C1—C2—C3	0.4 (3)	C22—C23—C24—C25	−0.1 (4)
C20—C1—C2—C3	−177.1 (3)	O1—C24—C25—C26	−178.5 (3)
C1—C2—C3—C4	1.6 (3)	C23—C24—C25—C26	0.3 (4)
C1—N1—C4—C5	−171.7 (3)	C24—C25—C26—C21	−0.4 (4)
Fe1—N1—C4—C5	−2.0 (4)	C22—C21—C26—C25	0.2 (4)
C1—N1—C4—C3	3.2 (3)	C5—C21—C26—C25	179.6 (3)
Fe1—N1—C4—C3	172.89 (18)	C11—C10—C28—C29	59.9 (3)
C2—C3—C4—N1	−3.0 (3)	C9—C10—C28—C29	−118.3 (3)
C2—C3—C4—C5	171.9 (3)	C11—C10—C28—C33	−121.7 (3)
N1—C4—C5—C6	−6.0 (4)	C9—C10—C28—C33	60.1 (3)
C3—C4—C5—C6	179.9 (3)	C33—C28—C29—C30	−1.8 (4)
N1—C4—C5—C21	171.6 (2)	C10—C28—C29—C30	176.8 (2)
C3—C4—C5—C21	−2.5 (4)	C28—C29—C30—C31	1.7 (4)
C9—N2—C6—C5	179.4 (3)	C34—O2—C31—C30	11.9 (4)
Fe1—N2—C6—C5	24.3 (4)	C34—O2—C31—C32	−168.6 (3)
C9—N2—C6—C7	0.5 (3)	C29—C30—C31—O2	178.7 (3)
Fe1—N2—C6—C7	−154.71 (18)	C29—C30—C31—C32	−0.8 (4)
C4—C5—C6—N2	−6.0 (4)	O2—C31—C32—C33	−179.4 (3)
C21—C5—C6—N2	176.4 (2)	C30—C31—C32—C33	0.1 (4)
C4—C5—C6—C7	172.8 (3)	C31—C32—C33—C28	−0.3 (4)
C21—C5—C6—C7	−4.8 (4)	C29—C28—C33—C32	1.1 (4)
N2—C6—C7—C8	−0.1 (3)	C10—C28—C33—C32	−177.4 (3)
C5—C6—C7—C8	−179.1 (2)	C14—C15—C35—C36	−57.4 (4)
C6—C7—C8—C9	−0.3 (3)	C16—C15—C35—C36	123.9 (3)
C6—N2—C9—C10	178.9 (2)	C14—C15—C35—C40	124.0 (3)
Fe1—N2—C9—C10	−25.6 (4)	C16—C15—C35—C40	−54.7 (3)
C6—N2—C9—C8	−0.6 (3)	C40—C35—C36—C37	−0.1 (4)
Fe1—N2—C9—C8	154.79 (17)	C15—C35—C36—C37	−178.6 (2)
C7—C8—C9—N2	0.6 (3)	C35—C36—C37—C38	−0.1 (4)
C7—C8—C9—C10	−179.0 (2)	C41—O3—C38—C37	−12.9 (4)
N2—C9—C10—C11	1.7 (4)	C41—O3—C38—C39	168.8 (2)
C8—C9—C10—C11	−178.8 (2)	C36—C37—C38—O3	−178.4 (2)
N2—C9—C10—C28	179.8 (2)	C36—C37—C38—C39	−0.2 (4)
C8—C9—C10—C28	−0.7 (4)	O3—C38—C39—C40	179.1 (2)
C14—N3—C11—C10	178.6 (2)	C37—C38—C39—C40	0.7 (4)
Fe1—N3—C11—C10	4.6 (4)	C38—C39—C40—C35	−0.9 (4)
C14—N3—C11—C12	−1.2 (3)	C36—C35—C40—C39	0.6 (4)
Fe1—N3—C11—C12	−175.27 (17)	C15—C35—C40—C39	179.3 (2)
C9—C10—C11—N3	9.8 (4)	C19—C20—C42—C43	−85.6 (4)
C28—C10—C11—N3	−168.3 (2)	C1—C20—C42—C43	91.5 (4)
C9—C10—C11—C12	−170.4 (2)	C19—C20—C42—C47	98.7 (4)
C28—C10—C11—C12	11.5 (4)	C1—C20—C42—C47	−84.2 (4)
N3—C11—C12—C13	0.0 (3)	C47—C42—C43—C44	1.2 (6)
C10—C11—C12—C13	−179.8 (3)	C20—C42—C43—C44	−174.8 (4)
C11—C12—C13—C14	1.2 (3)	C42—C43—C44—C45	0.3 (7)
C11—N3—C14—C15	−176.1 (2)	C43—C44—C45—O4	178.2 (3)
Fe1—N3—C14—C15	−2.2 (4)	C43—C44—C45—C46	−1.2 (6)
C11—N3—C14—C13	2.0 (3)	C48—O4—C45—C44	−6.2 (5)
Fe1—N3—C14—C13	175.89 (17)	C48—O4—C45—C46	173.2 (3)
C12—C13—C14—N3	−2.0 (3)	C44—C45—C46—C47	0.6 (5)
C12—C13—C14—C15	176.1 (3)	O4—C45—C46—C47	−178.8 (3)
N3—C14—C15—C16	0.0 (4)	C43—C42—C47—C46	−1.8 (5)
C13—C14—C15—C16	−177.8 (3)	C20—C42—C47—C46	174.0 (3)
N3—C14—C15—C35	−178.6 (2)	C45—C46—C47—C42	0.9 (5)
C13—C14—C15—C35	3.6 (4)	Fe1—O5—C49—C54	−86.9 (3)
C19—N4—C16—C15	−178.5 (3)	Fe1—O5—C49—C50	95.1 (3)
Fe1—N4—C16—C15	21.6 (4)	O5—C49—C50—F1	−1.2 (4)
C19—N4—C16—C17	1.7 (3)	C54—C49—C50—F1	−179.4 (3)
Fe1—N4—C16—C17	−158.28 (18)	O5—C49—C50—C51	176.4 (3)
C14—C15—C16—N4	−10.2 (4)	C54—C49—C50—C51	−1.7 (4)
C35—C15—C16—N4	168.4 (2)	F1—C50—C51—F2	0.8 (5)
C14—C15—C16—C17	169.6 (3)	C49—C50—C51—F2	−176.9 (3)
C35—C15—C16—C17	−11.8 (4)	F1—C50—C51—C52	−179.9 (3)
N4—C16—C17—C18	−0.6 (3)	C49—C50—C51—C52	2.5 (5)
C15—C16—C17—C18	179.5 (3)	F2—C51—C52—C53	178.2 (3)
C16—C17—C18—C19	−0.7 (3)	C50—C51—C52—C53	−1.2 (5)
C16—N4—C19—C20	174.4 (3)	C51—C52—C53—F3	178.6 (3)
Fe1—N4—C19—C20	−25.2 (4)	C51—C52—C53—C54	−0.8 (5)
C16—N4—C19—C18	−2.1 (3)	F3—C53—C54—F4	3.3 (4)
Fe1—N4—C19—C18	158.31 (18)	C52—C53—C54—F4	−177.3 (3)
C17—C18—C19—N4	1.8 (3)	F3—C53—C54—C49	−177.9 (3)
C17—C18—C19—C20	−174.7 (3)	C52—C53—C54—C49	1.5 (5)
N4—C19—C20—C1	1.1 (4)	O5—C49—C54—F4	0.5 (4)
C18—C19—C20—C1	177.1 (3)	C50—C49—C54—F4	178.6 (3)
N4—C19—C20—C42	178.0 (2)	O5—C49—C54—C53	−178.4 (3)
C18—C19—C20—C42	−6.1 (4)	C50—C49—C54—C53	−0.3 (4)
N1—C1—C20—C19	9.5 (4)	C6S—C1S—C2S—C3S	−44.3 (10)
C2—C1—C20—C19	−173.4 (3)	C1S—C2S—C3S—C4S	41.9 (12)
N1—C1—C20—C42	−167.3 (2)	C2S—C3S—C4S—C5S	−39.7 (12)
C2—C1—C20—C42	9.7 (4)	C3S—C4S—C5S—C6S	40.7 (11)
C4—C5—C21—C26	124.8 (3)	C4S—C5S—C6S—C1S	−44.7 (11)
C6—C5—C21—C26	−57.5 (4)	C2S—C1S—C6S—C5S	47.1 (11)
